# Atmosphere-Dependent Radiation Stabilization of Stearic Acid on Vaterite CaCO_3_: A Comparison of Gamma and Electron-Beam Irradiation

**DOI:** 10.3390/polym18070831

**Published:** 2026-03-28

**Authors:** Helena Biljanić, Urszula Gryczka, Marta Walo, Damir Kralj, Katarina Marušić

**Affiliations:** 1 Laboratory for Precipitation Processes, Division of Materials Chemistry, Ruđer Bošković Institute, Bijenička Cesta 54, 10 000 Zagreb, Croatia; hbachroj@irb.hr; 2 Institute of Nuclear Chemistry and Technology, Dorodna 16, 03-195 Warszaw, Poland; u.gryczka@ichtj.waw.pl (U.G.); m.walo@ichtj.waw.pl (M.W.)

**Keywords:** polymer filler, calcium carbonate, stearic acid, radiation crosslinking, gamma irradiation, electron-beam irradiation

## Abstract

Calcium carbonate is a widely used filler in polymer composites due to its low cost and ability to improve stiffness, dimensional stability, and impact resistance. However, its hydrophilic surface limits compatibility with nonpolar polymer matrices, making surface modification essential to improve filler dispersion and interfacial adhesion. Stearic acid is commonly applied as a surface modifier for calcium carbonate because it readily chemisorbs onto the mineral surface and forms densely packed self-assembled monolayers that improve hydrophobic character. Despite its widespread use, stearic acid exhibits limited thermal and interfacial stability under polymer processing conditions, motivating the development of stabilization strategies. In this work, gamma and electron-beam irradiation were applied to stearic-acid-modified calcium carbonate to modify the surface-bound stearic acid layer with the aim of enhancing its interfacial stability, surface resistance, and hydrophobic performance, and to evaluate the influence of irradiation atmosphere on these effects. The modified materials were characterized in terms of structural integrity, surface wettability, surface free energy, thermal stability, and optical properties. The results demonstrate that ionizing radiation enhances surface hydrophobicity and coating durability while preserving the crystal structure of the CaCO_3_ substrate. Gamma irradiation of stearic-acid-modified vaterite exhibited strong atmosphere dependence, with improved hydrophobicity under oxygen-free conditions, whereas electron-beam irradiation showed more robust and oxygen-insensitive behavior. Based on the observed improvements in hydrophobicity, surface free energy, and thermal stability, electron-beam irradiation emerges as a promising and less atmosphere-sensitive approach for producing durable stearic-acid-modified CaCO_3_ fillers suitable for polymer composite applications.

## 1. Introduction

Calcium carbonate (CaCO_3_) is one of the most widely used mineral fillers in the polymer and composite industries, with applications in plastics, rubbers, paints, sealants, and adhesives [1]. It exists in three anhydrous polymorphs: rhombohedral calcite, needle-like aragonite and metastable spherical vaterite, with calcite being the predominant commercial form [2]. The use of CaCO_3_ as a filler contributes to reduced production costs, improves the mechanical properties of polymer composites, and provides environmental benefits through partial replacement of the polymer matrix [3]. However, its hydrophilic surface limits compatibility with hydrophobic polymers such as polyethylene and polypropylene [4]. In addition, smaller particles tend to agglomerate due to their high surface energy and strong interparticle interactions [5]. To address these limitations, the surface of CaCO_3_ is commonly modified to improve dispersion and interfacial compatibility with nonpolar polymer matrices [1].

Surface modification of CaCO_3_ can be achieved through several approaches, including: (1) surfactant treatment with fatty acids; (2) coupling agents such as silanes, titanates, and aluminates; (3) unsaturated organic acids; (4) organic oligomer coatings; (5) water-soluble polymer modification; and (6) mechanochemical treatment via ball milling to enable in situ coating [5]. Among these methods, fatty acid modification is particularly attractive due to its simplicity, efficiency, and industrial relevance.

Fatty acids have the ability to adsorb onto the CaCO_3_ surface, forming organized surface layers [6]. Stearic acid, also known as octadecanoic acid, is the most widely used modifier owing to its low cost, commercial availability and strong interaction with CaCO_3_ surfaces [4,7,8,9,10,11,12,13]. The carboxyl group of stearic acid interacts with Ca^2+^ ions exposed at the CaCO_3_ surface through an acid–base reaction, leading to the formation of surface-bound calcium carboxylate species, while the long aliphatic chains orient outward, forming a dense hydrophobic interface [9,14,15]. This configuration reduces interparticle attraction, suppresses agglomeration, and improves dispersion in nonpolar polymer matrices [4,16].

However, these surface layers consist of low-molecular-weight organic molecules that are not interconnected through covalent bonds. As a result, their thermal and chemical stability may be limited, and they can be susceptible to desorption, molecular rearrangement, or changes in surface coverage under demanding processing conditions [17].

Exposing fatty acid layers to high-energy ionizing radiation can induce intermolecular coupling between adjacent aliphatic chains, resulting in the formation of a crosslinked polymer nanocoating [18]. Ionizing radiation thus represents an efficient strategy for stabilizing such molecular surface layers through covalent bond formation via radical polymerization. Free radicals react differently under gamma and electron-beam irradiation, largely because of differences in dose rate and the availability of oxygen. In the presence of oxygen, the high dose rate typical of electron-beam irradiation can favor crosslinking of carbon-centered radicals, whereas the lower dose rate of gamma irradiation allows more extensive reaction with oxygen to form peroxyl radicals, which may lead to oxidative degradation [19,20]. Compared to photochemical approaches such as UV irradiation, which often suffer from limited penetration depth and oxygen inhibition [21,22,23,24,25], high-energy radiation enables uniform energy deposition without the need for solvents, catalysts, or elevated temperatures [19,26]. This makes the process particularly attractive for scalable and environmentally benign surface modification of particulate materials.

Although fatty acid self-assembled monolayers are widely used to hydrophobize mineral fillers, additional stabilization is often required to ensure long-term performance under industrial processing conditions. Radiation-induced crosslinking offers a route to transform these molecular layers into mechanically robust polymer nanocoatings, thereby improving interfacial stability while preserving the simplicity of the original surface treatment approach.

Both gamma and electron-beam irradiation provide sufficient energy to initiate crosslinking reactions. Although gamma irradiation, which originates from radioactive isotopes such as ^60^Co, is less compatible with continuous industrial polymer processing, it enables homogeneous energy deposition and deep penetration, allowing uniform modification of thick and bulk materials [19]. In contrast, electron-beam irradiation, generated using electron accelerators, offers faster treatment rates, precise dose control, and straightforward integration into industrial production lines, albeit with more limited penetration depth [19,27]. The complementary characteristics of these irradiation techniques make their direct comparison particularly relevant for evaluating technological feasibility and scalability of radiation-induced surface crosslinking.

In our previous work, radiation-induced crosslinking of fatty acid monolayers on metallic substrates was successfully demonstrated, resulting in compact and stable polymer nanocoatings using gamma irradiation [18,28,29,30,31]. In this work, both gamma and e-beam irradiation were employed to evaluate their effectiveness in forming crosslinked nanocoatings on stearic-acid-modified CaCO_3_ and to assess their suitability for scalable industrial implementation. Vaterite was selected as a model CaCO_3_ polymorph due to its high specific surface area, enhanced surface reactivity, and predominantly spherical morphology. These characteristics facilitate efficient adsorption of surface modifiers and enable sensitive evaluation of radiation-induced surface crosslinking. Stearic acid was adsorbed onto the CaCO_3_ surface and exposed to high-energy ionizing radiation (gamma rays and e-beam) under oxygen-rich and oxygen-free conditions. The resulting structural, morphological, and surface property changes were systematically investigated.

## 2. Materials and Methods

### 2.1. Synthesis of Vaterite

Calcium carbonate polymorph vaterite was synthesized by mixing 50 mL of 0.33 mol/dm^3^ Na_2_CO_3_ (Kemika, Zagreb, Croatia) with 50 mL of 0.33 mol/dm^3^ CaCl_2_ (Merck, Darmstadt, Germany), followed by 90 s of sonication using a Branson Sonifier 250 ultrasonic homogenizer (Branson Ultrasonics, Danbury, CT, USA). The resulting precipitate was filtered through a 0.22 µm membrane, washed twice with deionized water and dried at 100 °C for one hour.

### 2.2. Self-Assembly of SA

Stearic acid (SA) (Kemika, Croatia) was adsorbed onto the washed CaCO_3_ precipitate by immersing it in 20 mL of 10 mmol/dm^3^ SA solution in ethanol (VWR, Leuven, Belgium) and stirring at room temperature using a magnetic stirrer IKA RCT Basic (IKA-Werke GmbH & Co. KG, Staufen, Germany). After 10 min, the mixture was filtered through a 0.22 µm membrane, washed twice with deionized water, and dried at 100 °C for one hour.

### 2.3. Irradiation of Samples

Different irradiation conditions were applied to induce crosslinking of SA adsorbed on the CaCO_3_ surface and to study the effects of ionizing radiation on untreated CaCO_3_. To compare the effects of different radiation sources on polymer nanocoating formation and material modification, both gamma and e-beam irradiation were employed under oxygen-rich and oxygen-free conditions.

#### 2.3.1. Gamma Irradiation

The samples were sealed in glass flasks, and prior to irradiation, half were purged with argon to obtain oxygen-free conditions, while the remaining samples were left in air to provide oxygen-rich conditions. Samples were irradiated at the Ruđer Bošković Institute in Zagreb, Croatia, using the panoramic gamma irradiation facility of the Laboratory for Radiation Chemistry and Dosimetry equipped with a ^60^Co source at room temperature. Dose mapping of the facility was performed using ionization chambers, ethanol-chlorobenzene dosimeters, and Monte Carlo simulation calculations [32,33]. Four absorbed doses (20, 40, 60, and 80 kGy) were administered at a dose rate of 12 kGy/h.

#### 2.3.2. E-Beam Irradiation

The samples were sealed in plastic bags and, prior to irradiation, half were purged with nitrogen to establish oxygen-free conditions, while the remaining samples were kept in air to provide oxygen-rich conditions. Electron-beam irradiation was performed at the Institute of Nuclear Chemistry and Technology in Warsaw, Poland, using an ILU-6 accelerator operating at an electron energy of 1.7 MeV at room temperature.

Absorbed doses of 20, 40, and 70 kGy were applied at a dose rate of 16 kGy/min, with a dose uniformity of ±10%. The delivered dose was verified using alanine dosimeters, analyzed by electron paramagnetic resonance (EPR) spectroscopy using an MS5000 spectrometer (Magnettech, Freiberg, Germany). The peak-to-peak amplitude of the central spectral line was used to quantify the dose.

### 2.4. Characterization of Materials

Fourier-transform infrared spectroscopy (FTIR-ATR) was conducted using a Bruker Tensor II spectrometer (Bruker Optik GmbH, Ettlingen, Germany) equipped with an attenuated total reflectance (ATR) module to identify functional groups on CaCO_3_ samples. FTIR spectra were recorded at room temperature over a wavenumber range of 400–3200 cm^−1^, with a resolution of 2 cm^−1^ and a total of 32 scans per measurement. Each sample was analyzed three times to ensure reproducibility and confirm the representative spectrum for each sample type.

Thermogravimetric analysis was performed using a TGA 4000 Thermogravimetric Analyzer (PerkinElmer, Shelton, CT, USA) under an inert nitrogen atmosphere. Measurements were performed over a temperature range of 30 to 900 °C at a heating rate of 10 °C/min.

Contact angle measurements were performed using a Contact Angle System OCA 20 goniometer (Data Physics, Filderstadt, Germany). A 1 μL droplet of water, diiodomethane, and ethylene glycol was applied for each measurement under ambient atmospheric conditions. Water was used to assess hydrophobicity, while diiodomethane and ethylene glycol were used to calculate surface energy. To ensure accuracy, each measurement was performed three times. Data Physics SCA20 Version 5.2 software was used for surface energy calculations.

Color measurements were conducted using a spectrophotometer model CM-2600d (Konica Minolta, Tokyo, Japan). Measurements were acquired over a 3 mm diameter area using diffuse illumination and an 8° viewing angle, with the CIE standard illuminant D65 representing average daylight. For each sample, four consecutive measurements were recorded and averaged to determine the color coordinates, and the procedure was repeated three times to ensure accuracy.

For contact angle and color measurements, samples were pressed into pellets using a 2-ton load applied for 1 min to obtain smooth and uniform surfaces.

## 3. Results and Discussion

To evaluate the role of oxygen on radiation-induced stabilization consistent with crosslinking of stearic acid (SA) adsorbed on CaCO_3_, gamma and e-beam irradiation were applied under oxygen-free and oxygen-rich conditions. The effects of ionizing radiation on both untreated CaCO_3_ particles and SA-modified CaCO_3_ were evaluated by analyzing structural stability, thermal behavior, optical properties, and surface wettability, thereby enabling assessment of CaCO_3_ stability and crosslinking efficiency of the surface-bound SA layer. Specifically, FTIR-ATR was used to assess structural stability of the CaCO_3_ substrate, TGA to evaluate thermal behavior, contact angle and surface energy measurements to probe surface modification, and colorimetric analysis to examine irradiation-induced optical changes.

### 3.1. Preparation and Surface Modification of CaCO_3_

CaCO_3_ was synthesized using ultrasonic agitation, resulting in porous submicron-sized structures. This approach is based on ultrasonically assisted precipitation from an aqueous solution containing calcium and carbonate ions. SA was introduced onto the CaCO_3_ surface through spontaneous self-assembly by immersing the synthesized particles in an ethanol-based SA solution, obtaining CaCO_3_+SA samples. While several preparation techniques, including co-precipitation, were evaluated, spontaneous self-assembly yielded the most effective surface coverage and was therefore selected for further investigations.

**Figure 1:**
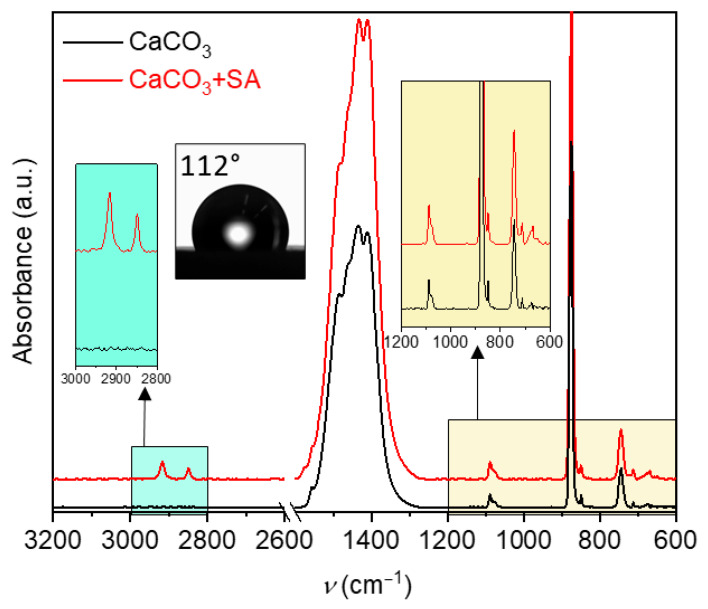
FTIR-ATR spectra of Calcium carbonate (CaCO_3_) synthesized by ultrasonic agitation and stearic-acid-modified CaCO_3_ (CaCO_3_+SA). For clarity, spectra are vertically offset. The inset shows a water contact angle measurement (112 ± 1°) on the CaCO_3_+SA surface.

To confirm the structure of the synthesized CaCO_3_ and verify SA adsorption on the CaCO_3_ surface, FTIR-ATR spectroscopy was employed. Figure 1 shows the FTIR-ATR spectrum of untreated CaCO_3_. Comparison with reference spectra of CaCO_3_ polymorphs revealed vibrational bands characteristic predominantly of the vaterite polymorph [34,35]. A distinct broad peak in the ν_3_ region (1400–1470 cm^−1^) is attributed to asymmetric C–O stretching vibrations, while the ν_1_ band at 1089 cm^−1^ corresponds to symmetric C–O stretching. A sharp, well-defined peak at 876 cm^−1^ (ν_2_) is associated with out-of-plane bending of the carbonate group, and the ν_4_ band observed at 745 cm^−1^ is related to in-plane bending of carbonate ions [35]. In addition, a weak band at approximately 712 cm^−1^ can be observed, indicating the presence of trace amounts of calcite, which is commonly detected in ultrasonically precipitated CaCO_3_ systems. For clarity, spectra are vertically offset.

Figure 1 also shows the FTIR-ATR spectrum of CaCO_3_ after SA adsorption. The SA adsorption on the CaCO_3_ surface is confirmed by the appearance of characteristic alkyl chain bands at 2850 cm^−1^ (symmetric) and 2917 cm^−1^ (antisymmetric) [36]. These band positions are indicative of highly ordered alkyl chains in an all-trans conformation, which is characteristic of densely packed and well-organized surface assemblies.

The absence of absorption bands at approximately 1700 and 935 cm^−1^, which are characteristic of the C=O stretching and O–H bending vibrations of protonated carboxylic acid groups, indicates that no free, non-bound SA is present on the surface [36].

Due to the highly hydrophilic nature of unmodified CaCO_3_, stable water droplets cannot be formed on the untreated mineral surface, preventing reliable contact angle measurements. After SA modification, stearate ions interact with surface Ca^2+^ sites, orienting their long aliphatic chains outward and thereby converting the surface from hydrophilic to hydrophobic [14]. Surface hydrophobicity, therefore, serves as a sensitive indicator of surface coverage and coating uniformity. Water contact angle measurements, which directly reflect surface wettability, were used to evaluate this behavior, with higher contact angle values corresponding to increased hydrophobicity. In the context of this study, surface hydrophobicity was selected as a primary functional parameter for assessing radiation-induced modification and stabilization of the stearic acid nanocoating. The inset in Figure 1 shows a representative water droplet on the CaCO_3_+SA surface with a contact angle of 112°, confirming successful hydrophobization and the formation of a densely packed organic monolayer.

### 3.2. Effects of Ionizing Radiation on Vaterite and SA Modified Vaterite

To investigate radiation-induced modifications of both the CaCO_3_ substrate and the surface-bound stearic acid layer, CaCO_3_ and CaCO_3_+SA samples were subjected to gamma and electron-beam irradiation under controlled atmospheric conditions, oxygen-rich and oxygen-free. For gamma irradiation, absorbed doses of 20, 40, 60, and 80 kGy were applied at a dose rate of 12 kGy/h, while for e-beam irradiation, doses of 20, 40, and 70 kGy were applied at a dose rate of 16 kGy/min.

#### 3.2.1. Structural Stability of CaCO_3_ Under Gamma and E-Beam Irradiation (FTIR)

**Figure 2:**
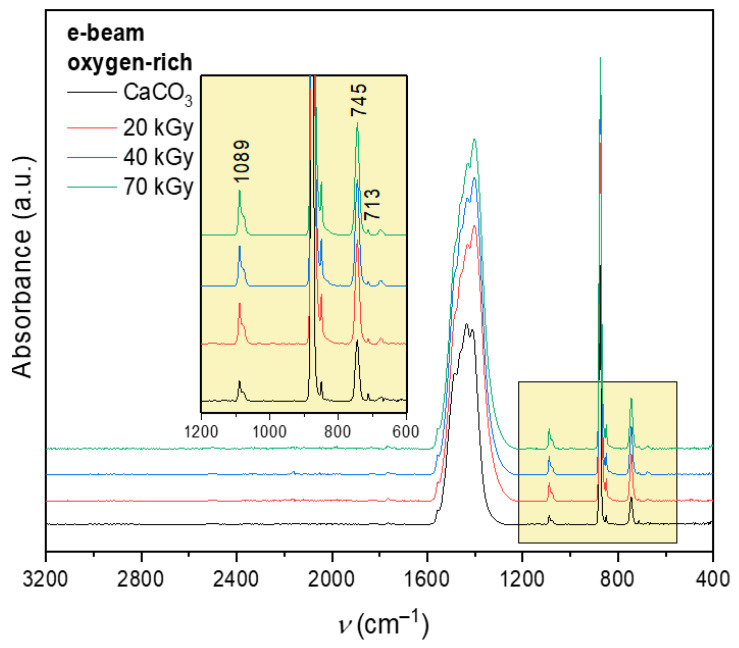
FTIR-ATR spectrum of CaCO_3_ after e-beam irradiation in oxygen-rich atmosphere. For clarity, spectra are vertically offset.

Figure 2 shows the FTIR-ATR spectrum of CaCO_3_ after e-beam irradiation under oxygen-rich conditions as a representative example. A comparison of CaCO_3_ samples irradiated by gamma and e-beam sources, under both oxygen-free and oxygen-rich atmospheres, revealed no observable differences within the resolution of the measurement. Therefore, only one representative spectrum is shown. The characteristic vaterite peak at 745 cm^−1^ remains clearly preserved in all samples. Moreover, no systematic change in the relative intensity of the calcite-related band at 712 cm^−1^ compared to the vaterite band at 745 cm^−1^ is observed, indicating that irradiation does not induce polymorphic transformation of CaCO_3_. For clarity, spectra are vertically offset.

The absence of structural changes in CaCO_3_ after irradiation is important for the interpretation of subsequent results, as it confirms that the observed thermal, optical, and surface property modifications originate primarily from radiation-induced reactions within the surface-bound SA layer rather than from alterations of the mineral substrate. Preservation of the vaterite structure also indicates that the applied irradiation doses are compatible with maintaining the structural integrity of the filler material.

#### 3.2.2. Radiation-Induced Optical Changes (Color Analysis)

**Figure 3:**
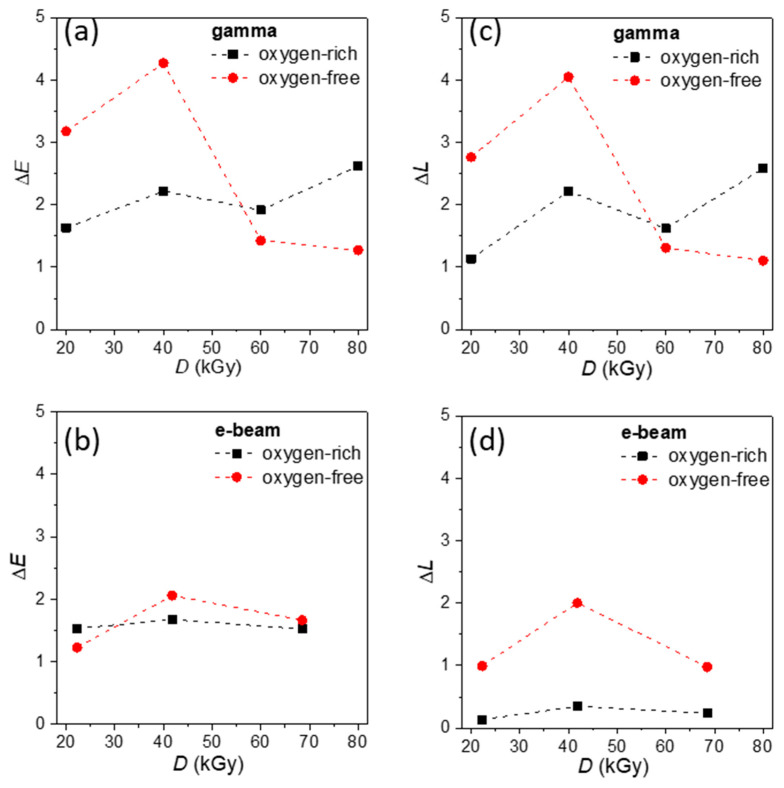
Radiation-induced color changes in CaCO_3_ as a function of absorbed dose: total color difference Δ*E* after (**a**) gamma, and (**b**) e-beam irradiation, as well as lightness change Δ*L* after (**c**) gamma and (**d**) e-beam irradiation.

Radiation-induced optical changes were evaluated first on untreated CaCO_3_ to assess whether irradiation alters the appearance of the mineral substrate itself, independent of the SA coating. The total color difference (Δ*E*) was used as a quantitative measure of overall color change as a function of dose and irradiation atmosphere (Figure 3a,b). Here, Δ*E* represents the total color difference, Δ*L* the change in lightness, while Δ*a* and Δ*b* describe changes along the green–red and blue–yellow color axes, respectively. Analysis of the individual color coordinates showed that the observed variations are mainly associated with changes in lightness (Δ*L*, Figure 3c,d), whereas the chromatic components (Δ*a* and Δ*b*) remain small and close to zero across the investigated dose range and thus are not presented. This indicates that irradiation primarily affects sample brightness rather than inducing pronounced hue shifts.

Colorimetric results show a clear dependence of Δ*E* on irradiation dose and atmospheric conditions (Figure 3a,b). Figure 3a presents the total color change after gamma irradiation, and it is evident that the atmosphere in which irradiation is performed significantly influences both the magnitude and evolution of color changes.

In an oxygen-free atmosphere, larger color changes are observed at lower doses (Δ*E* > 3 at 20–40 kGy), with a pronounced maximum at 40 kGy (Figure 3a). With further dose increase to 60–80 kGy, Δ*E* decreases to ~1.3–1.5, indicating that additional irradiation does not produce proportional optical changes. This type of behavior has also been reported for other gamma irradiated inorganic and composite materials and is commonly attributed to competing radiation-induced processes [37,38,39]. At lower doses, the formation of color centers and radiation defects dominates, leading to increased optical absorption and visible color change. At higher doses, defect recombination, saturation of available trapping sites, and partial radiation-induced annealing effects can occur, resulting in stabilization or partial compensation of radiation-induced defects. As a result, further dose increase does not produce proportional color changes, indicating that the optical response approaches a quasi-saturation regime at higher doses. Notably, the trend in Δ*E* is primarily governed by changes in lightness Δ*L* (Figure 3c).

In contrast, under oxygen-rich conditions, Δ*E* remains lower at low doses and changes more gradually with dose (Figure 3a), suggesting that oxygen moderates early defect build-up and shifts the balance toward slower, cumulative surface modifications.

The total color change after e-beam irradiation is shown in Figure 3b. Overall, Δ*E* values remain relatively low across the investigated dose range, indicating that e-beam treatment induces only moderate optical modification of CaCO_3_. Under oxygen-free conditions, Δ*E* increases up to 40 kGy and then slightly decreases at the highest applied dose, suggesting a weak saturation behavior of radiation-induced color changes. In an oxygen-rich atmosphere, the changes are less pronounced, with Δ*E* remaining nearly constant across the investigated dose range and showing only minor variations. The difference between the two atmospheres is relatively small, particularly at 70 kGy, indicating that the influence of oxygen on radiation-induced color change is considerably less pronounced for e-beam irradiation than for gamma irradiation. This behavior is consistent with the much higher dose rate of e-beam irradiation, which limits defect accumulation and oxygen diffusion effects, resulting in weaker atmosphere-dependent optical responses.

#### 3.2.3. Surface Wettability and Surface Energy

For polymer filler applications, surface hydrophobicity and surface energy represent the key functional parameters, as they directly influence filler–matrix compatibility, dispersion behaviour, and interfacial adhesion in nonpolar polymer systems. Since stearic acid modification is intended to convert the intrinsically hydrophilic CaCO_3_ surface into a polymer-compatible interface, changes in wettability provide a sensitive and application-oriented indicator of coating integrity and radiation-induced stabilization efficiency. In this study, surface properties were therefore used as a proxy for composite performance, enabling systematic evaluation of irradiation effects on the filler surface without introducing additional variables associated with polymer processing and formulation.

The irradiation dose ranges were selected based on a stepwise experimental approach. Gamma irradiation was initially applied using incremental dose steps of 20 kGy to systematically identify the dose range associated with favorable surface property changes and coating stabilization. Based on these results, a similar dose window was subsequently investigated using e-beam irradiation to enable direct comparison between the two radiation sources and to evaluate the feasibility of translating the optimized treatment conditions to a more industrially scalable process.

**Figure 4:**
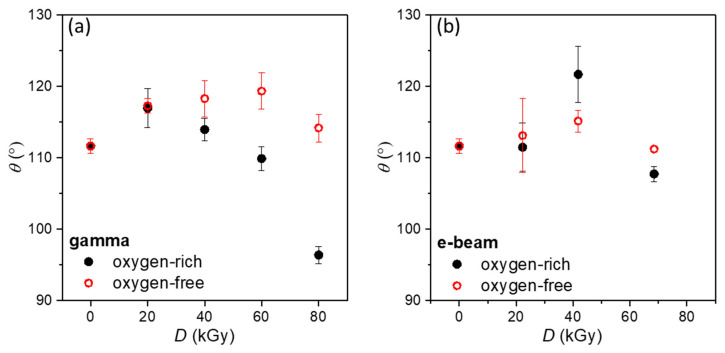
Water contact angle measurements of CaCO_3_+SA after: (**a**) gamma and (**b**) e-beam irradiation.

The hydrophobic behaviour of CaCO_3_+SA samples irradiated under oxygen-rich and oxygen-free atmospheres is shown in Figure 4. The non-irradiated CaCO_3_+SA sample exhibits a relatively high contact angle value, confirming the formation of a predominantly hydrophobic surface after stearic acid modification. Figure 4a presents the evolution of the water contact angle after gamma irradiation. Samples irradiated in an oxygen-rich atmosphere generally show lower contact angle values, which can be attributed to radiation-induced surface oxidation and partial degradation of the organic coating. In contrast, gamma irradiation under oxygen-free conditions results in increased contact angle values compared to the non-irradiated sample, with a pronounced maximum observed at 60 kGy. This behavior indicates enhanced stabilization of the stearic acid layer, consistent with radiation-induced intermolecular coupling and coating consolidation reported for fatty acid systems, leading to improved surface hydrophobicity.

Upon e-beam irradiation (Figure 4b), the contact angle exhibits only moderate variations with increasing dose. For the electron-beam experiments, absorbed doses of 20, 40, and 70 kGy were applied, selected to approximate the gamma irradiation dose range while accounting for accelerator operating constraints. Although the nominal dose values do not exactly match those used for gamma irradiation, the selected dose window enables identification of the functional optimum within the investigated range. In an oxygen-rich atmosphere, a slight increase in contact angle is observed, followed by a minor decrease at the highest dose. A similar trend is found under oxygen-free conditions, although the absolute values remain slightly lower than those obtained in the presence of oxygen. The relatively small differences between the two atmospheres indicate that oxygen plays a less pronounced role in influencing surface hydrophobicity during e-beam treatment compared to gamma irradiation.

This behavior is likely related to the substantially higher dose rate applied during e-beam irradiation (16 kGy/min) compared to gamma irradiation (12 kGy/h), which results in rapid energy deposition and short radical lifetimes. Under these conditions, the time available for oxygen diffusion and oxygen-mediated radical reactions is limited, thereby reducing the influence of the surrounding atmosphere on surface modification processes.

**Figure 5:**
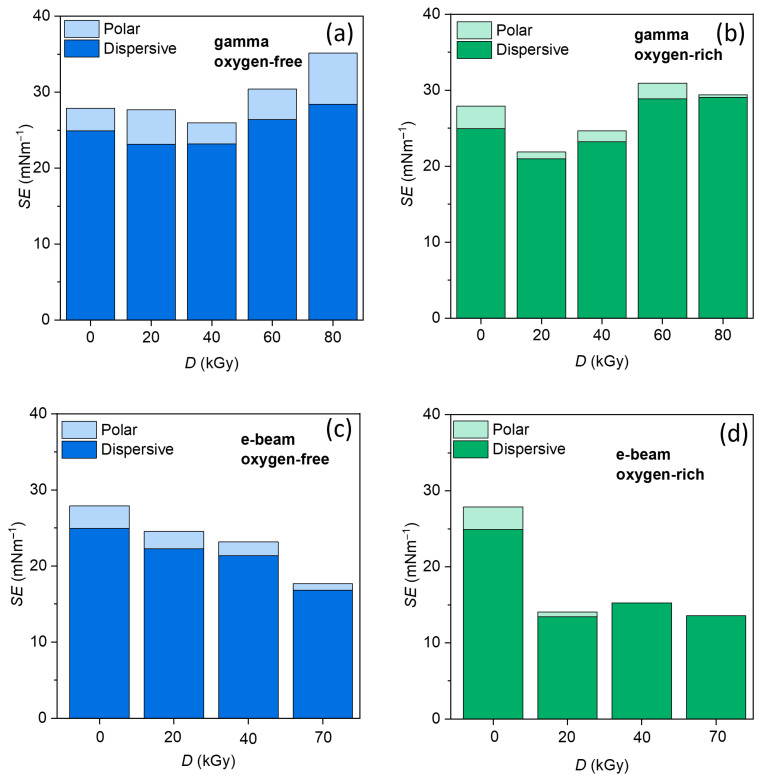
Surface energy of CaCO_3_+SA samples calculated from contact angle measurements after: (**a**) gamma irradiation in oxygen-free atmosphere, (**b**) gamma irradiation in oxygen-rich atmosphere, (**c**) e-beam irradiation in oxygen-free atmosphere, and (**d**) e-beam irradiation in oxygen-rich atmosphere.

To complement the contact angle results, surface free energy (SE) values were evaluated in order to quantify irradiation-induced changes in surface polarity and hydrophobic character (Figure 5). Across all samples, the total surface free energy is dominated by the dispersive component, while the polar contribution remains comparatively small, reflecting the predominantly nonpolar character of the stearic-acid-modified surface.

For gamma irradiation, distinct trends are observed depending on the irradiation atmosphere. Under oxygen-free conditions (Figure 5a), the total surface free energy initially decreases up to approximately 40 kGy, indicating enhanced surface hydrophobicity and coating stabilization. At higher doses (60 and 80 kGy), a pronounced increase in surface free energy is observed, suggesting radiation-induced modification of the stearic acid layer, including possible chain scission, oxidation, or partial loss of coating integrity, which results in increased surface polarity.

In contrast, gamma irradiation in an oxygen-rich atmosphere (Figure 5b) exhibits a markedly different behavior, characterized by a strong reduction in surface free energy at low dose (20 kGy), followed by an increase at intermediate doses and a slight decrease at the highest dose. Overall, the less stable and non-monotonic response observed under oxygen-rich conditions indicates that gamma irradiation in the presence of oxygen is less favorable for achieving consistent surface hydrophobicity and coating stabilization. This behavior reflects the competing effects of possible radiation-induced crosslinking and oxygen-mediated oxidative processes.

In the case of e-beam irradiation, samples treated under oxygen-free conditions (Figure 5c) exhibit a gradual and nearly monotonic decrease in total surface free energy with increasing dose, indicating progressive stabilization and hydrophobization of the surface layer. Under oxygen-rich conditions (Figure 5d), markedly different behavior is observed, characterized by a rapid and pronounced reduction in surface free energy already at the lowest applied dose (20 kGy), followed by minor variations at higher doses. Despite these different kinetic behaviors, both irradiation atmospheres result in similarly low surface free energy values at higher doses, indicating that e-beam irradiation effectively promotes surface hydrophobization regardless of oxygen presence. This reduced sensitivity to atmospheric oxygen is consistent with the high dose rate of e-beam irradiation, which limits oxygen diffusion and suppresses oxygen-mediated degradation pathways.

A direct comparison between irradiation techniques further reveals that e-beam treatment produces substantially lower surface free energy values than gamma irradiation, primarily due to a stronger reduction in the dispersive component while maintaining a minimal polar contribution. This combination indicates the formation of a more homogeneous, polymer-like surface and highlights e-beam irradiation as the more favorable approach for producing hydrophobic CaCO_3_ fillers with improved compatibility for nonpolar polymer composite applications [12].

#### 3.2.4. Thermal Stability of SA Coating (TGA)

**Figure 6:**
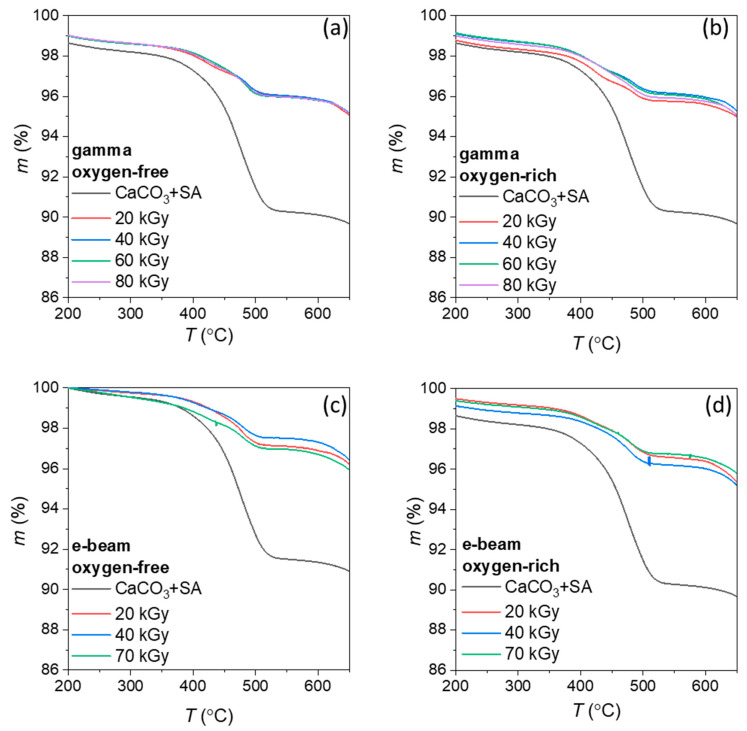
TGA curves of CaCO_3_+SA after: (**a**) gamma irradiation in oxygen-free atmosphere, (**b**) gamma irradiation in oxygen-rich atmosphere, (**c**) e-beam irradiation in oxygen-free atmosphere, and (**d**) e-beam irradiation in oxygen-rich atmosphere.

Thermogravimetric analysis curves of irradiated CaCO_3_+SA samples are shown in Figure 6. The unirradiated CaCO_3_+SA sample exhibits a pronounced mass loss in the temperature range of approximately 394–509 °C, which is mainly attributed to the removal of surface-bound stearic acid [6]. After irradiation, all samples display reduced mass loss in the stearic-acid-related temperature range and improved thermal stability of the organic layer. This behavior is consistent with radiation-induced stabilization, likely involving intermolecular crosslinking processes that enhance the thermal resistance of the surface-bound organic phase, as reported for radiation-treated organic layers [26].

When gamma irradiation was applied, improved thermal stability was observed in both oxygen-free and oxygen-rich atmospheres (Figure 6a,b), as evidenced by the reduced mass loss in the stearic-acid-related temperature range compared to the non-irradiated reference. Under oxygen-free conditions (Figure 6a), the irradiated samples exhibit slightly more uniform stabilization behavior across the investigated dose range. In an oxygen-rich atmosphere (Figure 6b), stabilization is also present, although minor variations between doses suggest the involvement of competing oxygen-mediated reactions that can influence the balance between crosslinking and degradation.

A similar behavior is observed for e-beam irradiation (Figure 6c,d), where all irradiated samples exhibit reduced coating-related mass loss in the stearic-acid-related temperature range compared to the non-irradiated reference, indicating improved thermal stability of the surface layer. However, the differences between the individual e-beam doses are minimal, and no clear dose-dependent trend can be resolved from the TGA curves alone.

Overall, the TGA results indicate enhanced thermal resistance of the surface-bound SA layer after irradiation, which is consistent with radiation-induced stabilization of the organic coating while preserving the structural integrity of the CaCO_3_ substrate.

The observed improvements in surface hydrophobicity, reduction in surface free energy, and enhanced thermal stability are consistent with radiation-induced stabilization of surface-bound stearic acid chains, likely involving intermolecular crosslinking. While direct spectroscopic confirmation of covalent bond formation was not performed in this study, the interpretation is supported by the observed functional property changes and by analogous behavior previously reported for fatty acid monolayers on metallic substrates under comparable irradiation conditions [18,30].

Together with the FTIR-ATR, colorimetric, contact angle, and surface energy results, the TGA data provide a more complete picture of the irradiation effects on the system. FTIR-ATR showed that the CaCO_3_ substrate remained structurally unchanged and did not undergo detectable polymorphic transformation, while colorimetric analysis revealed only limited changes in the appearance of untreated CaCO_3_. In contrast, contact angle and surface energy measurements demonstrated clear changes in surface properties after treatment, and TGA showed enhanced thermal resistance of the surface-bound SA layer. Taken together, these complementary results indicate that irradiation largely preserves the mineral substrate while affecting the behavior and stability of the surface-bound organic coating.

## 4. Conclusions

Gamma and e-beam irradiation were successfully applied to CaCO_3_ modified by stearic acid (SA) with the primary objective of enhancing and stabilizing surface hydrophobicity for improved compatibility with nonpolar polymer matrices. Both irradiation techniques preserved the structural integrity of the CaCO_3_ substrate, as confirmed by FTIR analysis, ensuring that the observed property changes originate from modification of the surface-bound organic layer. Contact angle and surface energy measurements demonstrate that irradiation effectively improves surface hydrophobicity, with the magnitude and stability of these effects strongly dependent on irradiation atmosphere and radiation source. Colorimetric analysis of untreated CaCO_3_ further confirmed that irradiation induces only moderate optical changes, indicating that substrate degradation does not limit the applicability of the treatment.

For SA-modified samples, clear differences between irradiation techniques were observed. Gamma irradiation produced the highest hydrophobicity enhancement under oxygen-free conditions, but exhibited pronounced sensitivity to atmospheric oxygen, leading to less stable surface behavior under air exposure. In contrast, e-beam irradiation resulted in consistently low surface free energy values under both oxygen-free and oxygen-rich conditions, indicating a more robust and atmosphere-independent surface modification. This reduced oxygen sensitivity is attributed to the high dose rate of e-beam irradiation, which limits oxygen diffusion and suppresses oxygen-mediated degradation pathways. Thermogravimetric analysis further confirmed improved thermal stability of the surface-bound stearic acid layer after irradiation, supporting radiation-induced stabilization of the coating.

The combined improvements in wettability, surface energy, and thermal resistance indicate effective radiation-induced stabilization of the stearic acid layer. Overall, e-beam irradiation emerges as the more technologically attractive approach due to its stable hydrophobization performance, reduced sensitivity to processing atmosphere, and suitability for continuous high-throughput treatment, making it particularly promising for large-scale production of hydrophobic CaCO_3_ fillers for polymer composite applications.

## Data Availability

The data presented in this study are available upon request from the corresponding authors.

## Abbreviations

The following abbreviations are used in this manuscript:

ATR	Attenuated total reflectance
CaCO_3_	Calcium carbonate
CIE	Commission Internationale de l’Éclairage (color system)
Δ*E*	Total color difference
EPR	Electron paramagnetic resonance
FTIR	Fourier-transform infrared spectroscopy
SA	Stearic acid
SE	Surface energy
TGA	Thermogravimetric analysis
Δ*a*, Δ*b*	Chromaticity coordinates
Δ*L*	Lightness difference
